# Cambrian origin of the CYP27C1-mediated vitamin A_1_-to-A_2_ switch, a key mechanism of vertebrate sensory plasticity

**DOI:** 10.1098/rsos.170362

**Published:** 2017-07-05

**Authors:** Ala Morshedian, Matthew B. Toomey, Gabriel E. Pollock, Rikard Frederiksen, Jennifer M. Enright, Stephen D. McCormick, M. Carter Cornwall, Gordon L. Fain, Joseph C. Corbo

**Affiliations:** 1 Department of Integrative Biology and Physiology, University of California Los Angeles, Los Angeles, CA 90095, USA; 2 Department of Ophthalmology and Jules Stein Eye Institute, University of California Los Angeles, Los Angeles, CA 90095, USA; 3 Department of Pathology and Immunology, Washington University School of Medicine, St Louis, MO 63110, USA; 4 Department of Physiology and Biophysics, Boston University School of Medicine, Boston, MA 02118, USA; 5 Conte Anadromous Fish Research Laboratory, US Geological Survey, Leetown Science Center, Turners Falls, MA 01370, USA

**Keywords:** visual ecology, photoreceptor, *Petromyzon marinus*

## Abstract

The spectral composition of ambient light varies across both space and time. Many species of jawed vertebrates adapt to this variation by tuning the sensitivity of their photoreceptors via the expression of CYP27C1, an enzyme that converts vitamin A_1_ into vitamin A_2_, thereby shifting the ratio of vitamin A_1_-based rhodopsin to red-shifted vitamin A_2_-based porphyropsin in the eye. Here, we show that the sea lamprey (*Petromyzon marinus*), a jawless vertebrate that diverged from jawed vertebrates during the Cambrian period (approx. 500 Ma), dynamically shifts its photoreceptor spectral sensitivity via vitamin A_1_-to-A_2_ chromophore exchange as it transitions between photically divergent aquatic habitats. We further show that this shift correlates with high-level expression of the lamprey orthologue of CYP27C1, specifically in the retinal pigment epithelium as in jawed vertebrates. Our results suggest that the CYP27C1-mediated vitamin A_1_-to-A_2_ switch is an evolutionarily ancient mechanism of sensory plasticity that appeared not long after the origin of vertebrates.

## 1. Introduction

Variability in the intensity and spectral composition of light in the natural world presents fundamental challenges for vision, and many features of the vertebrate eye are adaptations to meet these challenges. In some aquatic environments such as ponds, streams and rivers, specific wavelengths of light are scattered or absorbed by sediment and dissolved organic matter, resulting in a marked shift in the spectral composition of light towards longer wavelengths [1,2]. A wide variety of aquatic organisms optimize visual sensitivity and discrimination by matching the sensitivities of their photoreceptors to the available light spectrum [2–5]. To maintain this match, as light environments change over space and time, many species have evolved mechanisms of visual system plasticity: that allow them to shift photoreceptor sensitivity dynamically. For example, some fish species switch the expression of opsin genes to match their visual sensitivity to ambient wavelengths (e.g. [6,7]). In this study, we focus on another widespread mechanism of visual system plasticity: the ‘rhodopsin–porphyropsin’ switch [8].

**Figure 1.**
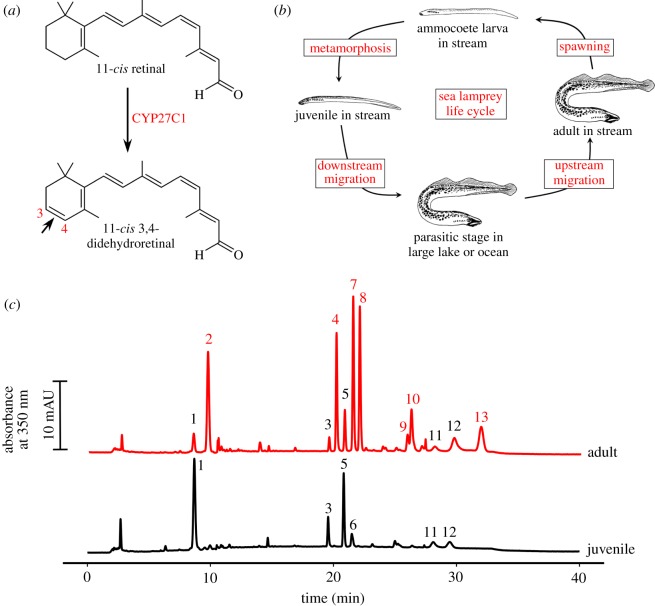
The eyes of adult upstream migrant lamprey are enriched with vitamin A_2_ chromophore. (*a*) In teleost fish and amphibians, CYP27C1 mediates the conversion of 11-*cis* retinal (a derivative vitamin A_1_) to 11-*cis* 3,4-didehydroretinal (a derivative of vitamin A_2_) to red-shift photoreceptor spectral sensitivities. (*b*) The sea lamprey moves between streams and large lakes/ocean through the course of its life cycle. (*c*) Representative HPLC chromatograms of retinoid extracts from juvenile and adult lamprey eyes. 3,4-Didehydroretinoids, unique to the adult eye, are numbered in red, and other retinoids, common to both adult and juvenile, are numbered in black. These peaks were identified based on comparisons to pure standards, published accounts, and spectral characteristics: 1, retinal ester; 2, 3,4-dehydroretinal ester; 3, *syn*-11-*cis*-retinal oxime; 4, *syn*-11-*cis*-3,4-dehydroretinal oxime; 5, *syn*-all-*trans*-retinal oxime; 6, *syn*-9-*cis*-retinal oxime; 7, *syn*-all-*trans*-3,4-dehydroretinal oxime; 8, *syn*-9-*cis*-3,4-dehydroretinal oxime; 9, *anti*-11-*cis*-3,4-dehydroretinal oxime; 10, 11-*cis*-3,4-dehydroretinol; 11, *anti*-all-*trans*-retinal oxime; 12, all-*trans*-retinol; 13, all-*trans*-3,4-dehydroretinol (peak spectra are given in electronic supplementary material, figure S1, and further details about the peaks are presented in electronic supplementary material, table S1).

Anadromous species such as Pacific salmon (*Oncorhynchus* sp.) spend most of their adult lives in the ocean where short-wavelength (blue–green) light predominates, but migrate to spawn in inland waterways enriched for long-wavelength (yellow–red) light [9]. To accommodate this shift in the light spectrum, salmon red-shift the sensitivity of their photoreceptors by switching their visual pigment chromophore from 11-*cis* retinal (a derivative of vitamin A_1_) to 11-*cis* 3,4-Didehydroretinal (a derivative of vitamin A_2_; figure 1*a*) [8,9]. This shift in the visual system is sometimes referred to as the ‘rhodopsin–porphyropsin’ switch, because vitamin A_1_-based photopigments have traditionally been called ‘rhodopsins’, whereas the red-shifted vitamin A_2_-based photopigments are collectively referred to as ‘porphyropsins’ [8]. Chromophore switching allows for adaptation of the visual system to changing spectral conditions and is used by a diversity of fishes, amphibians and reptiles [8,10–12]. Recently, the cytochrome P450 family member CYP27C1 was identified as the enzyme that mediates the conversion of vitamin A_1_ to A_2_ in both fish and amphibians [13]. This discovery raises the question of when this mechanism of sensory plasticity first arose during the course of vertebrate evolution.

Lampreys are jawless (agnathan) vertebrates which, together with hagfish, constitute the cyclostomata, the only surviving group from the earliest branch in the vertebrate subphylum. The lineage giving rise to cyclostomes diverged from the one giving rise to jawed vertebrates during the late Cambrian period about 500 Ma [14]. Like salmon, many lamprey species are anadromous: they begin life in freshwater streams as ammocoete larvae, and after several years undergo metamorphosis to become juveniles. The juveniles subsequently migrate either to large lakes or to the sea before returning to streams or rivers to spawn [15] (figure 1*b*). Earlier experiments showed that sea lamprey (*Petromyzon marinus*) can shift the retinoid content of their photoreceptors from vitamin A_1_ to A_2_ upon migration to their breeding grounds as adults [16,17]. This finding raises the intriguing possibility that a CYP27C1-mediated vitamin A_1_-to-A_2_ switch may have already been present in the last common ancestor of lampreys and jawed vertebrates. To investigate this possibility, we examined the physiology and spectral sensitivity of the photoreceptors of downstream migrating juvenile and upstream migrating adult lamprey and examined how these measures correlated with the vitamin A_1_ and A_2_ composition of the eye and the expression of *CYP27C1.*

## 2. Results

### 2.1. The eyes of upstream migrating adult lamprey contain vitamin A_2_

We sampled lamprey from a recently established land-locked population that spends the parasitic stage of its life cycle in Lake Huron instead of the ocean. To confirm that this population maintains the A_1_-to-A_2_ switch, we analysed the retinoid content of the eyes of juvenile and adult lamprey with high-performance liquid chromatography (HPLC). Consistent with coastal populations [16,17], the eyes of downstream migrating juveniles contained only vitamin A_1_ derivatives, while the eyes of adult lampreys contained both vitamin A_1_ and A_2_ derivatives, with the various forms of vitamin A_2_ making up approximately 82% of the total retinoid content (figure 1*c*; electronic supplementary material, table S1).

### 2.2. The physiological responses of juvenile lamprey photoreceptors are similar to those of adults

**Figure 2.**
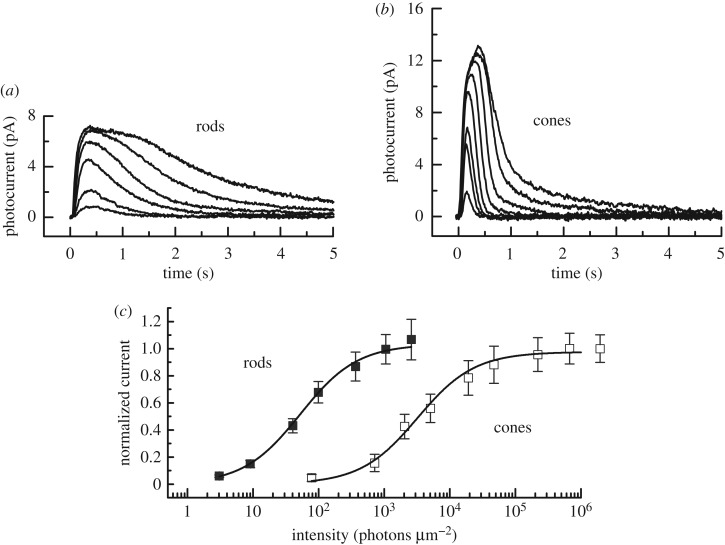
Current responses and sensitivity of juvenile lamprey rod and cone photoreceptors to brief light stimuli. (*a*) Mean responses of 13 rods to 20 ms 500 nm flashes given at *t* = 0 at the following intensities (in photons μm^−2^): 9, 40, 98, 366, 1055 and 2591. (*b*) Mean responses of six cones to 20 ms 600 nm flashes given at *t* = 0 at the following intensities (in photons µm^−2^): 78, 711, 2051, 5036, 1.91 × 10^4^, 2.20 × 10^5^, 6.71 × 10^5^ and 6.96 × 10^5^. (*c*) Normalized mean current response amplitudes (±s.e.) as a function of flash intensity for rods (closed squares) and cones (open squares). The same cells as in (*a*) and (*b*). Data for both cell types were fitted with the Michaelis–Menton equation, *r*/*r*_max_ = *I*/(*I* + *I*_½_), where *r*/*r*_max_ is the normalized current amplitude, *I* the flash intensity and *I*_½_ the flash intensity producing a half-maximal response. Best-fitting values of *I*_½_ were 52 photons μm^−2^ for rods and 3210 photons μm^−2^ for cones.

Juvenile lampreys are much smaller than adults, with eyes only about 20% as large in diameter, and the physiology of their photoreceptors has not been previously investigated. To investigate the physiological responses of the photoreceptors of juvenile lamprey, we used suction-electrode recording to measure the responses of single photoreceptors. Responses of juvenile photoreceptors were very similar to those of adults [18] (figure 2*a*,*b*). When we plotted the peak amplitude of the light response for the two photoreceptor types as a function of stimulus intensity (figure 2*c*), juvenile rods were approximately 85-fold more sensitive than cones, comparable to the value we obtained for adults [18]. Juvenile rod responses decay more slowly than cone responses, and single photons produce a response of about 0.4 pA, again very similar to the behaviour of adults photoreceptors [18,19] (electronic supplementary material, figure S2). Thus, the physiological properties of juvenile rods and cones showed no significant differences from those of their adult counterparts.

### 2.3. The spectral sensitivity of the rods and cones of adult migrant lamprey are red-shifted relative to those of the juvenile

**Figure 3.**
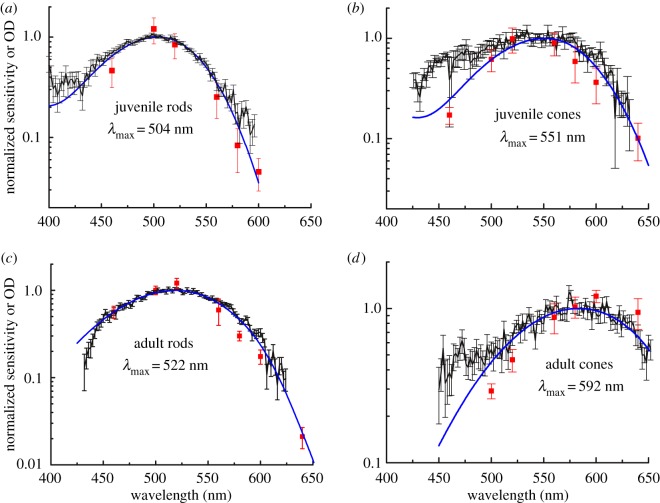
The photoreceptor spectral sensitivities of adult upstream migrant lamprey are long-wavelength shifted compared with those of juvenile downstream migrants. Normalized absorption spectra and spectral sensitivities for each condition are superimposed in a single graph. Black data points and black connecting lines represent microspectrophotometric determinations of mean pigment absorbance (±s.e.) normalized to absorbance at *λ*_max_, taken from 58 juvenile rods (*a*), nine juvenile cones (*b*), 70 adult rods (*c*) and 23 adult cones (*d*). Red data points represent the normalized mean spectral sensitivities (± s.e.) from suction-electrode recordings of nine juvenile rods (*a*), seven juvenile cones (*b*), 13 adult rods (*c*) and 14 adult cones (*d*). Blue curves for (*a*) and (*b*) are A_1_ visual-pigment nomograms [20] for best-fitting *λ*_max_'s of 504 nm for rods and 551 for cones; or weighted averages of A_1_ and A_2_ nomograms for adult photoreceptors (*c*,*d*) with best-fitting values of 18% A_1_ and 82% A_2_ for rods (*c*) and 14% A_1_ and 86% A_2_ for cones (*d*). The A_1_/A_2_ mixture modelling approach was adapted from Kefalov *et al*. [21].

To determine whether the differences in retinoid content between juvenile and adult lampreys are reflected in the spectral sensitivities of individual rods and cones, we recorded the responses of these cells to 460, 500, 520, 560, 580, 600 and 620 nm light. The results of these measurements are presented for juvenile rods and cones (red squares in figure 3*a*,*b*) and for adult rods and cones (red squares in figure 3*c*,*d*). In separate experiments, we used microspectrophotometry (MSP) to measure the mean normalized pigment absorbance of both juvenile and adult rods and cones, and we superimposed these data on top of the spectral sensitivities (black points in figure 3*a*,*d*). We found that juvenile rods have peak sensitivity and absorbance (*λ*_max_) at 504 nm, while adult rods have their *λ*_max_ at 522 nm (cf. figure 3*a*,*c*). By contrast, juvenile cones have *λ*_max_ = 551 nm, whereas adult cones have *λ*_max_ = 592 nm (cf. figure 3*b*,*d*). Thus, the spectral sensitivities of adult rods and cones are red-shifted relative to those of the juvenile.

To investigate the potential role of chromophore exchange in mediating the shift of rod and cone sensitivities to longer wavelengths in the adult, we fitted the MSP data with curves representing contributions from vitamin A_1_-based pigment only in juvenile lampreys, and a mixture model that incorporates contributions from pigments based on both vitamin A_1_ and vitamin A_2_ in the adult [20] (blue lines, figure 3). The data from juvenile rods and cones show a good fit to a pure vitamin A_1_-based template (figure 3*a*,*b*). By contrast, the visual pigments of adults are best fit by a weighted sum of pigment curves with 82% (rod) and 86% (cone) vitamin A_2_-based pigment, with the remaining fractions attributable to vitamin A_1_-based pigment (figure 3*c,d*). Additionally, the differing magnitudes of the spectral sensitivity shifts in the rods (Δ18 nm) versus cones (Δ41 nm) are consistent with the known dependence of the A_1_-to-A_2_ spectral shift on the *λ*_max_ of the opsin [22,23]. Thus, single-photoreceptor electrophysiological measurements and HPLC analyses both indicate the long-wavelength shift in sensitivity between juvenile and adult lamprey is the result of switching from a vitamin A_1_-based chromophore to a predominantly vitamin A_2_-based chromophore [17].

### 2.4. The accumulation of vitamin A_2_ in the adult retina is correlated with *CYP27C1* expression

Next, we asked if this A_1_-to-A_2_ chromophore shift in lamprey is mediated by a homologue of the enzyme CYP27C1, which was recently shown to catalyse the conversion of vitamin A_1_ into vitamin A_2_ in teleost fish and amphibians [13]. A *CYP27C1* orthologue had not been identified in any agnathan species to date as the 3,4-retinoid dehydrogenase responsible for the synthesis of vitamin A_2_. Therefore, we searched for an orthologue in sea lamprey by using polymerase chain reaction (PCR) with degenerate primers based on alignments of the amino acid sequences of known CYP27C1 homologues from multiple vertebrate species. In this way, we were able to amplify a portion of the *CYP27C1* transcript from adult sea lamprey eye cDNA (GenBank accession number MF163257). This transcript is predicted to encode a protein with 54% amino acid identity to zebrafish CYP27C1 (NP_001106808.2, AA100-538; electronic supplementary material, figure S3). Bioinformatic searches (BLASTP, Genbank) confirmed that, among all known and predicted sea lamprey protein sequences, this transcript shares the highest per cent identity with zebrafish CYP27C1.

In zebrafish (*Danio rerio*) and bullfrogs (*Lithobates catesbeianus*), CYP27C1 is specifically expressed in the retinal pigment epithelium (RPE) of the eye, and transcript levels are positively correlated with the presence of vitamin A_2_ [13]. Therefore, we predicted that adult lamprey, which have high levels of vitamin A_2_ in their eyes (figure 1*c*), would also show high levels of *CYP27C1* expression, specifically in the RPE.

**Figure 4.**
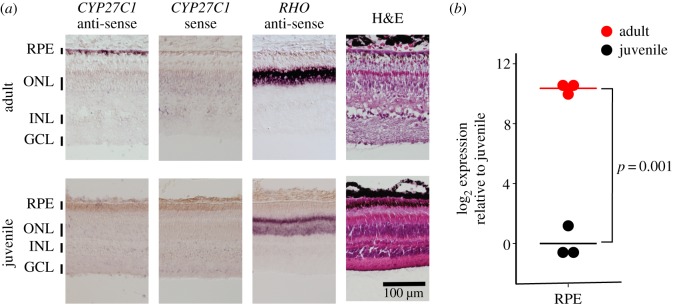
*CYP27C1* is specifically expressed in the RPE of adult upstream migrant lamprey. (*a*) ISH of cross sections of retina and RPE from juvenile and adult lamprey. The *CYP27C1* antisense probe labels the *CYP27C1* transcript in the RPE of adults; only weak background signal was observed in the negative control (*CYP27C1* sense probe), and the *Rhodopsin* (*RHO*) antisense probe (positive control) labels *RHO* in the ONL of both adults and juveniles. RPE, retinal pigment epithelium; ONL, outer nuclear layer; INL, inner nuclear layer; GCL, ganglion cell layer; H&E, haematoxylin and eosin staining. (*b*) qPCR analyses indicate that *CYP27C1* transcript levels are significantly higher in the RPE of adult lamprey compared with juveniles.

To investigate the expression of *CYP27C1* in lamprey, we performed *in situ* hybridization (ISH) and quantitative PCR (qPCR) to compare the eyes of juveniles and adults. Consistent with our prediction, *CYP27C1* is specifically expressed in the RPE of the adult upstream migrant and was not detectable in the juvenile (figure 4*a*). These qualitative differences were confirmed by qPCR analyses demonstrating that *CYP27C1* is expressed at more than 1000-fold higher levels in adult RPE compared with juvenile RPE (Welch's *t*-test: *t* = −16.29, d.f. = 2.43, *p* = 0.0014; figure 4*b*). These results strongly suggest that CYP27C1 mediates formation of vitamin A_2_ in the eyes of adult upstream migrant lamprey.

## 3. Discussion

Our study provides the first electrophysiological characterization of the rod and cone photoreceptors of juvenile lamprey and indicates that this developmental stage possesses receptors that are physiologically similar to those of adult lamprey [18,19]. The spectral sensitivities of photoreceptors from upstream adults were however significantly red-shifted compared with the downstream juveniles. Consistent with earlier studies of lamprey visual pigments [16,17], we found that this red-shift is attributable to a switch in the visual pigment chromophore from 11-*cis* retinal (A_1_) to 11-*cis* 3,4-didehydroretinal (A_2_). Concomitant with the vitamin A_1_-to-A_2_ shift, we observed a dramatic increase in the expression of *CYP27C1* in the RPE of adult lamprey compared with juveniles. Taken together, our data suggest that CYP27C1-mediating chromophore switching is an evolutionarily ancient mechanism of sensory plasticity.

Teleost fish and amphibians produce vitamin A_2_ in the eye through the controlled expression of CYP27C1 in the RPE [11,13]. Similarly, we find that *CYP27C1* is highly expressed in the RPE of adult upstream migrant lamprey that use the vitamin A_2_ chromophore. Previous biochemical analyses of adult lamprey indicated that vitamin A_2_ is found only in the eye [17]. Therefore, the same pattern of tissue-specific expression of CYP27C1 seen in teleost fish and amphibians likely mediates the production of vitamin A_2_ in the eyes of adult lamprey and may be widespread among lampreys. Recent transcriptomic analysis of the eyes of another lamprey species (*Geotria australis*) shows a similar pattern of elevated *CYP27C1* expression in upstream migrating adults (electronic supplementary material, figures S3 and S4) [24]. Thus, CYP27C1-mediated A_1_-to-A_2_ switching may be a mechanism of sensory plasticity conserved across a diversity of vertebrates.

Vertebrates evolved in the ocean and subsequently colonized freshwater habitats [25]. The light environment of fresh inland waterways, especially rivers and streams, is often turbid and red-shifted due to the presence of suspended sediments and dissolved organic matter. This turbidity likely represented a significant challenge to the visual systems of early vertebrates attempting to colonize such habitats. We therefore conclude that the CYP27C1-mediated vitamin A_1_-to-A_2_ switch in the eyes of early vertebrates may have been among the suite of adaptations that facilitated the early invasion of brackish and freshwater habitats.

## 4. Material and methods

### 4.1. Animals

Juvenile and adult sea lamprey (*P. marinus*) were provided to us by the Hammond Bay Biological Station of the US Geological Survey, Millersburg, MI, USA. Juvenile lamprey were captured by drift net in the St Marys River while in the process of migrating downstream to Lake Huron to begin the parasitic stage of the life cycle. Adult lamprey were captured in tributaries of Lake Huron (Ocqueoc River and Cheboygan River) in the process of their upstream spawning migration. Additional sea lamprey samples used for preliminary PCR analysis were obtained from the Connecticut River and reared at the Conte Anadromous Fish Research Laboratory in Turner Falls, MA, USA. Lamprey were kept in well-aerated tanks in cyclic 12 L/12 D h lighting in accordance with the rules and regulations of the NIH guidelines for research animals, as approved by the institutional animal care and use committee of the University of California, Los Angeles.

### 4.2. Analyses of retinoid content

We used HPLC to examine the retinoid content of eyes of juvenile and adult lamprey. The eyes were homogenized under dim light conditions in cold saline with a glass dounce. Retinaldehydes were then derivatized by treatment with hydroxylamine (Sigma, 255580) and extracted with hexane. The extract was dried under a stream of nitrogen, resuspended in 120 µl of hexane, and 100 µl was injected into an Agilent 1100 series HPLC equipped with a Zorbax RX-SIL column (4.6 × 250 mm, 5 µm, Agilent). The samples were eluted with a gradient mobile phase consisting of 0.5% ethyl acetate in hexane for 5 min then a ramp up to 10% ethyl acetate in hexane from 5 to 20 min, followed by isocratic conditions through 35 min. The column was held at 25°C, and the flow rate was 1.4 ml min^−1^ throughout the run. The samples were monitored with a photodiode array detector at 325, 350 and 380 nm, and retinoids were putatively identified by comparison to authentic standards or published accounts (electronic supplementary material, table S1) [26–29].

### 4.3. Single-cell spectral sensitivity and microspectrophotometry

Electrical responses of juvenile lamprey photoreceptor outer segments were recorded with suction electrodes as previously described for adult animals [18]. Light from a halogen lamp was passed through narrow band-pass interference filters and neutral absorption filters to provide stimuli of varying wavelengths and intensities. Spectral sensitivity was calculated cell by cell as the light required to elicit a criterion response at each tested wavelength and was normalized to the wavelength of peak sensitivity. MSP measurements of pigment absorbance were made as previously described for mouse rods [30], except that the measuring beam was confined to single lamprey rod or cone outer segments. The fitting of absorbance measurements to pigment nomograms was done for juvenile rods and cones with nomograms of Govardovskii *et al*. [20], and for adult rods and cones as in Kefalov *et al*. [21] with a weighted sum of nomograms by means of a program in R computing language (available in the accompanying Dryad repository: dx.doi.org/10.5061/dryad.kv3j7). Only the longer-wavelength α-bands of the nomograms were used for fitting, because absorbance at shorter wavelengths was complicated by scattering, the occurrence of pigment β-band absorption and the presence of photoproducts.

### 4.4. Analyses of *CYP27C1* expression

A partial sequence of the sea lamprey orthologue of *CYP27C1* was obtained via degenerate PCR. PCR primers (electronic supplementary material, table S2) were designed with the CODEHOP algorithm [31]; the input to the algorithm consisted of an alignment of the amino acid sequences of CYP27C1 from *D. rerio* (NP_001106808.2), *Xenopus tropicalis* (CAJ83899.1), *Chelonia mydas* (XP_007069504.1), *Apteryx australis* (XP_013816080.1), *Monodelphis domestica* (XP_001377301.3) and *Callithrix jacchus* (XP_002749285.1). Total RNA was extracted from adult RPE to generate cDNA as described below. PCR was performed with the Phusion Hot Start polymerase (NEB, M0536) following the manufacture's recommendations, and the resultant amplicons were sequenced via the Sanger method. Based on these initial sequencing results, additional PCR primers were designed (electronic supplementary material, table S2), and RACE PCR [32,33] was performed in an effort to obtain a full-length transcript.

*CYP27C1* transcript levels in RPE of juvenile and adult lamprey were measured by qPCR. Total RNA was extracted from the RPE of four juvenile and four adult individuals with Trizol reagent (Invitrogen, 10296010) and used to generate cDNA with the SuperScript IV reverse transcriptase (ThermoFisher, 18090010) following the manufacturer's protocols. Primers corresponding to the 3′ portion of the coding sequences of *CYP27C1* and *GAPDH* were selected (electronic supplementary material, table S2). *CYP27C1* primer efficiency was determined by assaying a dilution series of mature lamprey RPE cDNA. The primers produced a single amplicon as indicated by melt curve analyses, and were 96% efficient at the threshold (*C*_t_) levels used for quantification in the sample analyses. Measurements were made in three biological replicates with the Sybr^®^ Green PCR master mix (Life Technologies, 4309155) and the Applied Biosystems StepOne real-time PCR system. The technical replicates of individual samples were averaged and expression was compared (Δ*C*_t_) relative to *GAPDH.*

The localization of the *CYP27C1* transcript within the eyes of juvenile and adult lamprey was evaluated by ISH. The coding sequences of *CYP27C1* and *Rhodopsin* (*RHO*) obtained from mature lamprey RPE cDNA were cloned by blunt-ended ligation into the BlueScript vector pBSK+ (electronic supplementary material, table S2) and then used as templates for synthesis of digoxigenin-labelled probes following established methods [34]. Whole eyes of juvenile and adult lamprey were fixed in 4% paraformaldehyde in phosphate-buffered saline (PBS) overnight and then embedded in Tissue-Tek OCT compound (Sakura). Twelve micrometre horizontal sections through the centre of each eye were then prepared. Each section was incubated overnight at 4°C with 30% hydrogen peroxide in PBS in order to bleach the melanin pigmentation of the RPE, which can obscure the ISH signal. The sections were then hybridized, developed and mounted as previously described [34].

## Supplementary Material

Supplemental Material

## Data accessibility

The data supporting the findings of this study are available in the electronic supplementary material and the Dryad Digital Repository (http://dx.doi.org/10.5061/dryad.kv3j7) [35].

## References

[1] Jerlov NG 1976 Marine optics. Amsterdam, The Netherlands: Elsevier Scientific.

[2] Jokela-Määttä M, Smura T, Aaltonen A, Ala-Laurila P, Donner K 2007 Visual pigments of Baltic Sea fishes of marine and limnic origin. Vis. Neurosci. 24, 389–398. (doi:10.1017/S0952523807070459) 17822578 10.1017/S0952523807070459

[3] Lythgoe JN 1979 The ecology of vision. Oxford, UK: Clarendon Press.

[4] Lythgoe JN 1984 Visual pigments and environmental light. Vision Res. 24, 1539–1550. (doi:10.1016/S0042-6989(84)80003-6) 6398560 10.1016/s0042-6989(84)80003-6

[5] Cronin TW, Johnsen S, Marshall NJ, Warrant EJ 2014 Visual ecology. Princeton, NJ: Princeton University Press.

[6] Cheng CL, Novales Flamarique I 2004 Opsin expression: new mechanism for modulating colour vision. Nature 428, 279 (doi:10.1038/428279a) 10.1038/428279a 15029185

[7] Temple SE, Veldhoen KM, Phelan JT, Veldhoen NJ, Hawryshyn CW 2008 Ontogenetic changes in photoreceptor opsin gene expression in coho salmon (*Oncorhynchus kisutch*, Walbaum). J. Exp. Biol. 211, 3879–3888. (doi:10.1242/jeb.020289) 19043060 10.1242/jeb.020289

[8] Bridges CDB 1972 The rhodopsin–porphyropsin visual system. In Handbook of sensory physiology VII (ed. Dartnall HJA), pp. 417–480. Berlin, Germany: Springer.

[9] Beatty DD 1966 A study of the succession of visual pigments in Pacific salmon (*Oncorhynchus*). Can. J. Zool. 44, 429–455. (doi:10.1139/z66-045) 5932749 10.1139/z66-045

[10] Toyama M et al. 2008 Presence of rhodopsin and porphyropsin in the eyes of 164 fishes, representing marine, diadromous, coastal and freshwater species—a qualitative and comparative study. Photochem. Photobiol. 84, 996–1002. (doi:10.1111/j.1751-1097.2008.00344.x) 18422881 10.1111/j.1751-1097.2008.00344.x

[11] Reuter TE, White RH, Wald G 1971 Rhodopsin and porphyropsin fields in the adult bullfrog retina. J. Gen. Physiol. 58, 351–371. (doi:10.1085/jgp.58.4.351) 5315587 10.1085/jgp.58.4.351 PMC2226032

[12] Provencio I, Loew ER, Foster RG 1992 Vitamin A2-based visual pigments in fully terrestrial vertebrates. Vision Res. 32, 2201–2208. (doi:10.1016/0042-6989(92)90084-V) 1287997 10.1016/0042-6989(92)90084-v

[13] Enright JM et al. 2015 Cyp27c1 red-shifts the spectral sensitivity of photoreceptors by converting vitamin A1 into A2. Curr. Biol. 25, 3048–3057. (doi:10.1016/j.cub.2015.10.018) 26549260 10.1016/j.cub.2015.10.018 PMC4910640

[14] Kuraku S, Kuratani S 2006 Time scale for cyclostome evolution inferred with a phylogenetic diagnosis of hagfish and lamprey cDNA sequences. Zool. Sci. 23, 1053–1064. (doi:10.2108/zsj.23.1053) 17261918 10.2108/zsj.23.1053

[15] Hardisty MW 2006 Lampreys: life without jaws. Ceredigion, UK: Forrest Text.

[16] Crescitelli F 1956 The nature of the lamprey visual pigment. J. Gen. Physiol. 39, 423–435. (doi:10.1085/jgp.39.3.423) 13286458 10.1085/jgp.39.3.423 PMC2147542

[17] Wald G 1957 The metamorphosis of visual systems in the sea lamprey. J. Gen. Physiol. 40, 901–914. (doi: 10.1085/jgp.40.6.901) 13439167 10.1085/jgp.40.6.901 PMC2147579

[18] Morshedian A, Fain GL 2015 Single-photon sensitivity of lamprey rods with cone-like outer segments. Curr. Biol. 25, 484–487. (doi:10.1016/j.cub.2014.12.031) 25660538 10.1016/j.cub.2014.12.031 PMC4334710

[19] Asteriti S, Grillner S, Cangiano L 2015 A Cambrian origin for vertebrate rods. eLife 4, e07166 (doi:10.7554/eLife.07166) 10.7554/eLife.07166 PMC4502669 26095697

[20] Govardovskii VI, Fyhrquist N, Reuter T, Kuzmin DG, Donner K 2000 In search of the visual pigment template. Vis. Neurosci. 17, 509–528. (doi:10.1017/S0952523800174036) 11016572 10.1017/s0952523800174036

[21] Kefalov VJ, Estevez ME, Kono M, Goletz PW, Crouch RK, Cornwall MC, Yau KW 2005 Breaking the covalent bond—a pigment property that contributes to desensitization in cones. Neuron 46, 879–890. (doi:10.1016/j.neuron.2005.05.009) 15953417 10.1016/j.neuron.2005.05.009 PMC2885911

[22] Parry JW, Bowmaker JK 2000 Visual pigment reconstitution in intact goldfish retina using synthetic retinaldehyde isomers. Vision Res. 40, 2241–2247. (doi:10.1016/S0042-6989(00)00101-2) 10927111 10.1016/s0042-6989(00)00101-2

[23] Harosi FI 1994 An analysis of two spectral properties of vertebrate visual pigments. Vision Res. 34, 1359–1367. (doi:10.1016/0042-6989(94)90134-1) 8023444 10.1016/0042-6989(94)90134-1

[24] Lamb TD, Patel H, Chuah A, Natoli RC, Davies WI, Hart NS, Collin SP, Hunt DM 2016 Evolution of vertebrate phototransduction: cascade activation. Mol. Biol. Evol. 33, 2064–2087. (doi:10.1093/molbev/msw095) 27189541 10.1093/molbev/msw095 PMC4948711

[25] Halstead LB 1985 The vertebrate invasion of fresh-water. Phil. Trans. R. Soc. Lond. B 309, 243–258. (doi:10.1098/rstb.1985.0085)

[26] Babino D, Perkins BD, Kindermann A, Oberhauser V, von Lintig J 2015 The role of 11-cis-retinyl esters in vertebrate cone vision. FASEB J. 29, 216–226. (doi:10.1096/fj.14-261693) 25326538 10.1096/fj.14-261693 PMC4285540

[27] Kane MA, Napoli JL 2010 Quantification of endogenous retinoids. Methods Mol. Biol. 652, 1–54. (doi:10.1007/978-1-60327-325-1_1) 20552420 10.1007/978-1-60327-325-1_1 PMC4113000

[28] Zonta F, Stancher B 1984 High-performance liquid chromatography of retinals, retinols (vitamin A1) and their dehydro homologues (vitamin A2): improvements in resolution and spectroscopic characterization of the stereoisomers. J. Chromatogr. 301, 65–75. (doi:10.1016/S0021-9673(01)89179-2) 6501494 10.1016/s0021-9673(01)89179-2

[29] Landers GM, Olson JA 1988 Rapid, simultaneous determination of isomers of retinal, retinal oxime and retinol by high-performance liquid chromatography. J. Chromatogr. 438, 383–392. (doi:10.1016/S0021-9673(00)90269-3) 3384888 10.1016/s0021-9673(00)90269-3

[30] Nymark S, Frederiksen R, Woodruff ML, Cornwall MC, Fain GL 2012 Bleaching of mouse rods: microspectrophotometry and suction-electrode recording. J. Physiol. 590, 2353–2364. (doi:10.1113/jphysiol.2012.228627) 22451436 10.1113/jphysiol.2012.228627 PMC3424757

[31] Rose TM, Henikoff JG, Henikoff S 2003 CODEHOP (COnsensus-DEgenerate hybrid oligonucleotide primer) PCR primer design. Nucleic Acids Res. 31, 3763–3766. (doi:10.1093/nar/gkg524) 12824413 10.1093/nar/gkg524 PMC168931

[32] Scotto-Lavino E, Du G, Frohman MA 2006 3′ end cDNA amplification using classic RACE. Nat. Protoc. 1, 2742–2745. (doi:10.1038/nprot.2006.481) 17406530 10.1038/nprot.2006.481

[33] Scotto-Lavino E, Du G, Frohman MA 2006 5′ end cDNA amplification using classic RACE. Nat. Protoc. 1, 2555–2562. (doi:10.1038/nprot.2006.480) 17406509 10.1038/nprot.2006.480

[34] Enright JM, Lawrence KA, Hadzic T, Corbo JC 2015 Transcriptome profiling of developing photoreceptor subtypes reveals candidate genes involved in avian photoreceptor diversification. J. Comp. Neurol. 523, 649–668. (doi:10.1002/cne.23702) 25349106 10.1002/cne.23702 PMC4367231

[35] Morshedian A, Toomey MB, Pollock GE, Frederiksen R, Enright JM, McCormick SD, Cornwall MC, Fain GL, Corbo JC 2017 Data from: Cambrian origin of the CYP27C1-mediated vitamin A_1_-to-A_2_ switch, a key mechanism of vertebrate sensory plasticity. Dryad Digital Repository. (http://dx.doi.org/10.5061/dryad.kv3j7) 10.1098/rsos.170362 PMC5541561 28791166

