# Supplementary material for: Cambrian origin of the CYP27C1-mediated vitamin A1-to-A2 switch, a key mechanism of vertebrate sensory plasticity
Source: R Soc Open Sci. 2017 Jul 5;4(7):170362. doi: 10.1098/rsos.170362 (PMC5541561; doi:10.1098/rsos.170362)
Supplement: Supplemental Material [file rsos170362supp1.pdf]

Supplemental Material

Morshedian et al. Cambrian origin of the CYP27C1-mediated vitamin A_1-to-A_2 switch, a key mechanism of vertebrate sensory plasticity

Figure S1. The UV-Vis spectra of the retinoids measured by HPLC of juvenile and adult lamprey eyes. Spectra correspond to numbered peaks presented in Figure 1 of main text and table S1.

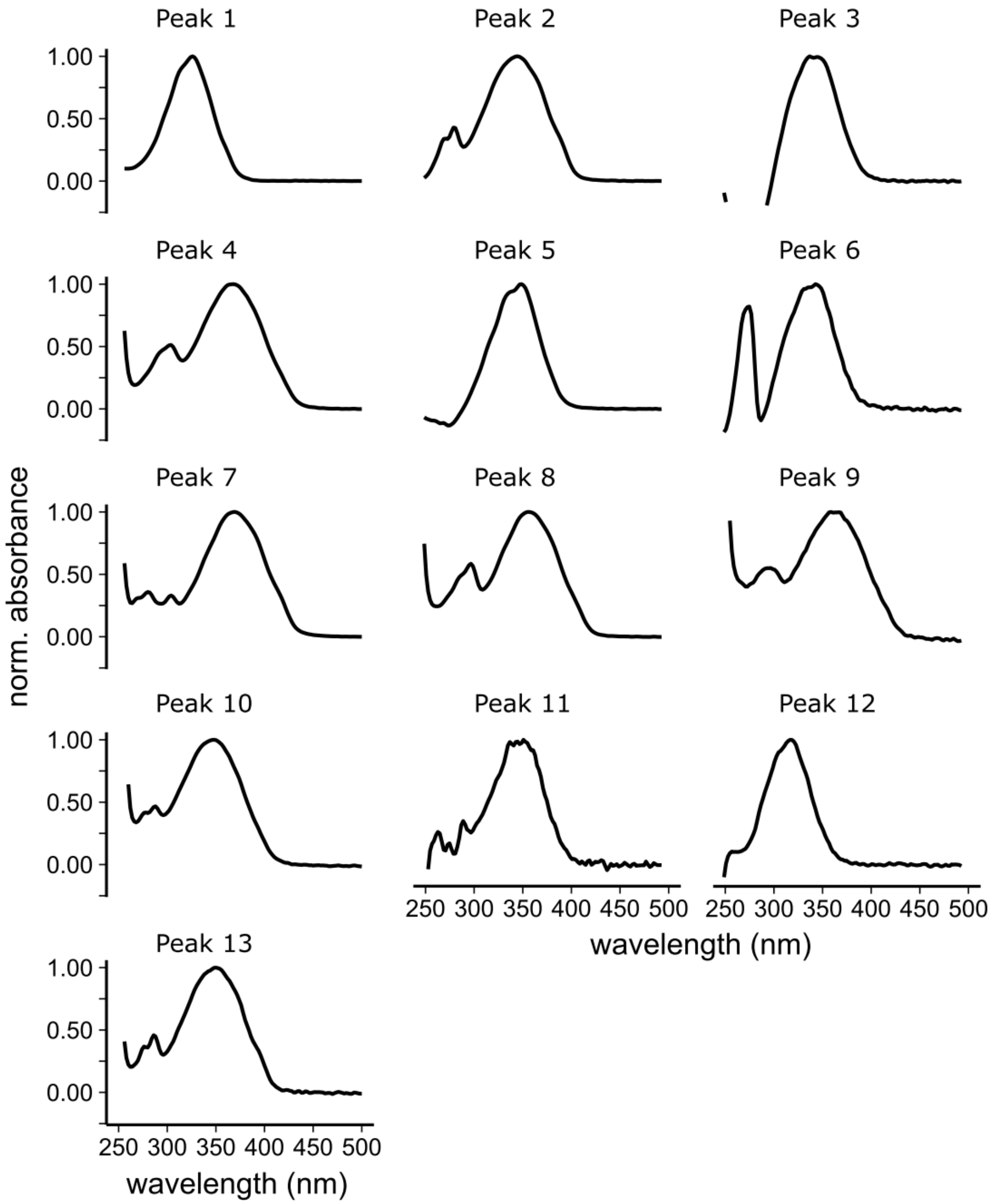

Figure S2. Response decay of juvenile rod and cone photoreceptors and single-photon responses of juvenile lamprey rods. *(a)*, Normalized mean light response of 13 rods and 6 cones to 20-ms flashes. Flash intensities were 98 photons μm^{-2} for rods and 2051 photons μm^{-2} for cones. Decay phases of both responses were fitted with a single exponential decay function with a time constant of recovery (τ_{REC}) of 564 ms for rods and 97 ms for cones. The recordings were made from the same cells as in figure 2 of main text. *(b)*, Mean single-photon response of juvenile rods, calculated for 9 rods from the squared mean and variance (as in Morshedian & Fain [16]).

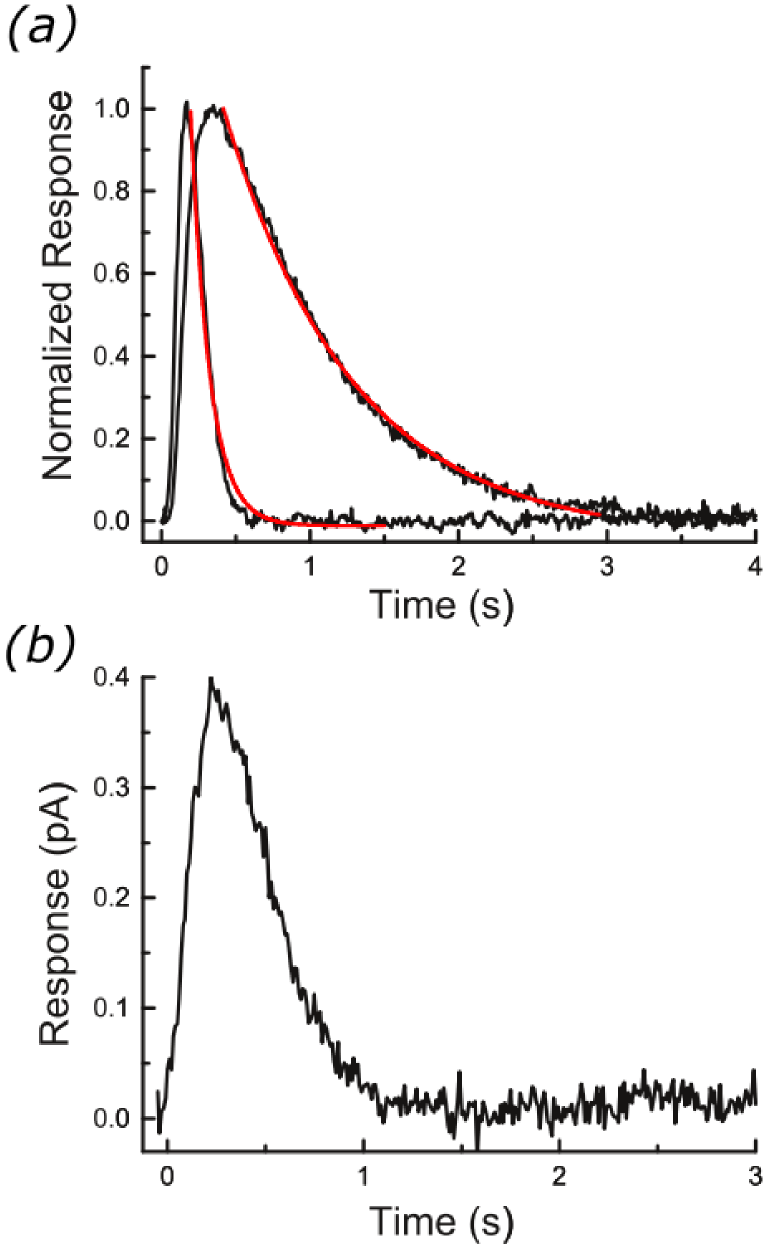

Figure S3. A linear alignment of the amino acid sequence of the *Petromyzon marinus* CYP27C1 ortholog with those of the pouched lamprey (*Geotria australis*) and zebrafish (*Danio rerio*). "*" indicates identity between the species, and "." indicates similarity (PAM 250 matrix analysis). The *Geotria australis* transcript was assembled as described in the legend of figure S4.

```
D. rerio       1 MALQSTILHMARKNLLQESCRQLLIQTHGLHKSVASGSLEIAAHSQADLKEESAVSPAEE
P. marinus     1 ------------------------------------------------------------
G. australis   1 ------------------------------------------------------------

D. rerio      61 VQKAARVKSLKEMPGPSTVANLLEFFYRDGFSRIHEIQMEHAKKYGKIFKSRFGPQFVVS
P. marinus     1 ---------------------------GSPPTGGTKKGNQEAKRFGAGWGTSFGPQFVVS
G. australis   1 -------------------------------------------MWGTSFGPQFMVS
                                                .         ..... .....*****.**

D. rerio     121 IADRDMVAQVLRSESATPQRGNMESWKEYRDLRGRSTGLISAEGDEWLKMRSVLRQLIMR
P. marinus    34 LASPGLVAHVLRSEGPAPQRANMASWREYRALRGRANGLISTEGEEWLRMRAVLRQPLMR
G. australis  14 LASPALVAHVLRSEGPAPQRSNMASWHEYRVLRGRANGLISAEGEEWLRMRAVLRQPLMR
                 .*....**.*****...***.**.**.*** ****..****.**.***.**.****..**

D. rerio     181 PRDVAVFSSDVNDVVADLVKRVKTLRSQQDDSQTVLNINDLFFKYAMEGVATILYETRLG
P. marinus    94 ARSVWRHSEQINAIVEDVVSRVRCDRDE--RDGTVRNVNGLLFKFAMEGMASVLFERRLG
G. australis   74 ARSVWRHADDINAIVDDVVTRVRLDRDA--RTATVTNVNGLLFKFAMEGMASILFERRLG
                 .*.*.......*..*.*.*.**. .*.   ...** *.*.*.**.****.*..*.*.***

D. rerio     241 CLENEIPKMSQEYITALHLMFSSFKTTMYAGAIPKWLRPIIPKPWEEFCSSWDGLFKFSQ
P. marinus   152 CLAPEVPADTRDYIAALQLMFSMFKTTMYAGAIPHWLRPVLPGPWEDFCHSWDGLFRFSE
G. australis 132 CLAAEVPADTRDYIAALQLMFSMFKTTMYAGAIPRWLRPVLPGPWEDFCRSWDGLFHFSE
                 **. *.*.....**.**.****.***********.****..*.***.**.******.**.

D. rerio     301 IHVDKRLSEIKKQMEKS--EEIKGGLLTHMLVTREMNLEEIYANMTEMLLAGVDTTSFTL
P. marinus   212 IHVDARARELEEERATGGAARGQAGFLTEQLMSGALSRQELYANVTEMLLAGVDTTSFTM
G. australis 192 KHVDARARELEEERVSGRRA-REPGFLSEQLASRALSREELYANVTEMLLAGVDTTSFTL
                 .***.*..*..... ..  .   .*.*...*........*.***.**************.

D. rerio     359 SWSTYLLARHPTIQQQIFEEVDR--VLGGRVPTGEDVPYLPLIRGLVKETLRLFPVLPGN
P. marinus   272 SWCLDLLARHPLTQEAVLREVRERGPSGGWAPSAEHVAEMPLLRGVLKETLRLYPVLPGN
G. australis 251 SWCLELLARHPATQEAVLREVRQRGPSGVGPPSAEHVAEMPLLRGVLKETLRLYPVLPGN
                 **...****** .*.....**. ....*.  *..*.*...**.**..******.******

D. rerio     417 GRVTHDDLIVGGYLIPKGTQLALCHYSTSMDEENFPRPEEFRPDRWIRKDASD-------
P. marinus   332 GRVTQTDMVLGGHHIPKGTQLAMCHYSTSHDPATFPQPESFRPERWLRDGASRGGPAGPH
G. australis 311 GRVTQTDMVLGGHHIPNGTQLALCHYSTSHDPAAFPHPESFRPERWLRDGSSASGGDYDD
                 ****..*...**..**.*****.******.*.. **.**.***.**.*...*  .

D. rerio     470 ----------------------------RVDNFGSIPFGYGIRSCIGRRIAELEMHLALTQ
P. marinus   392 GDGSGEGEEEGEEEAAHRCPESAHESWHSQGFASIPFGYGVRSCIGRRVAELEIHLALAR
G. australis 371 -------GGDAEQAAARPCPATAHAGQHAQGFASIPFGYGVRSCVGRRVAELEIHLALAR
                          ...  ... .. ...   . ..*.*******.***.***.****.****..

D. rerio     503 LLQNFHIEVSPQTTEVHAKTHGLLCPGASINLRFTDRK-
P. marinus   452 LLLEFRLEVLPGAPRVPAKTHGLLGPGGSIDLRFVDLS-
G. australis 424 LLLEFRVEAVPGAPPVPAKTHGLLGPGASMDLRFVDRRS
             541 **..*..*..*... *.*******.**.*..***.*..
```

Figure S4. The eyes of upstream migrant *Geotria australis* have elevated *CYP27C1* transcript levels compared to downstream migrants. RNA-seq data for *Geotria australis* (derived from a recent publication [19]) were acquired from the NCBI Short Read Archive (SRR214917, SRR214918, SRR214919). These data were used as input to the Trinity algorithm[1] to generate a *de novo* transcript assembly. An ortholog of *CYP27C1* was identified with a translated nucleotide (tblastn) search of the assembly using *Danio rerio* CYP27C1 as a query (NP_001106808.2) (figure S3). RNA-seq sequence reads for upstream and downstream migrants were aligned to the assembly and expression was quantified with RSEM[2]. Read counts are presented as fragments per kilobase of transcript per million mapped reads (FPKM). Given the limited sample size (two upstream replicates but only one downstream), we did not carry out any further statistical analyses.

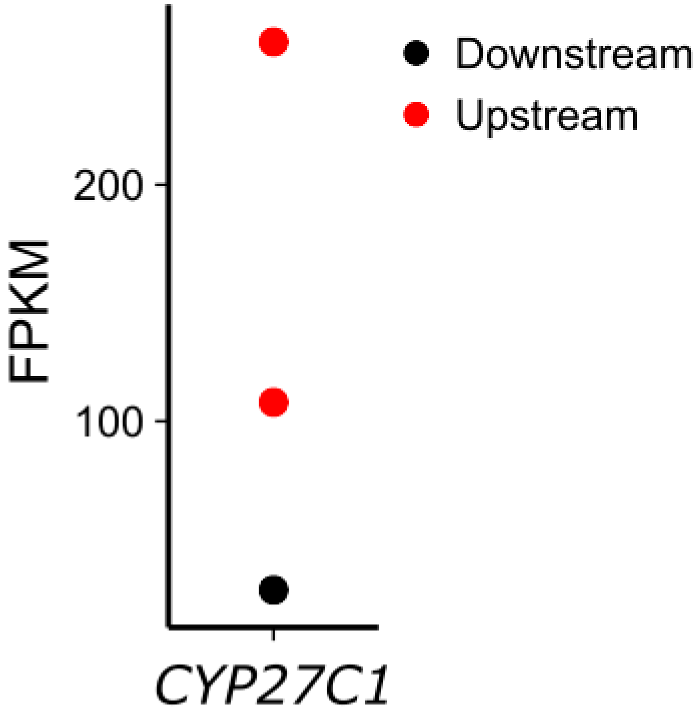

1. Haas, B.J. et al. De novo transcript sequence reconstruction from RNA-seq using the Trinity platform for reference generation and analysis. Nat Protoc 8, 1494-512 (2013).
2. Li, B. & Dewey, C.N. RSEM: accurate transcript quantification from RNA-Seq data with or without a reference genome. BMC Bioinformatics 12, 323 (2011).

Table S1. The identity and amounts of specific retinoids measured by HPLC of juvenile and adult lamprey eyes. These data correspond to the numbered peaks presented in Figure 1 and S1.

Retinoid	Proportion of downstream juvenile profile	Proportion of upstream adult profile
Peak 1 - retinal ester	0.61	0.05
Peak 2 - 3,4-dehydroretinal ester	-	0.17
Peak 3 - *syn*-11-*cis*-retinal oxime	0.06	0.01
Peak 4 - *syn*-11-*cis*-3,4-dehydroretinal oxime	-	0.13
Peak 5 - *syn*-all-trans-retinal oxime*	0.16	0.05
Peak 6 - *syn*-9-*cis*-retinal oxime	0.04	-
Peak 7 - *syn*-all-trans-3,4-dehydroretinal oxime	-	0.17
Peak 8 - *syn-9-cis*-3,4-dehydroretinal oxime	-	0.17
Peak 9 - *anti*-11-*cis*-3,4-dehydroretinal oxime	-	0.03
Peak10 - 11-*cis*-3,4-dehydroretinol	-	0.08
Peak 11 - *anti*-all-trans-retinal oxime*	0.03	0.01
Peak 12 - all-trans-retinol*	0.10	0.05
Peak 13 - all-trans-3,4-dehydroretinol*	-	0.08

* identified by comparison to pure standards, other peaks were identified by comparison to published reports[1-4]

1. Babino, D., Perkins, B.D., Kindermann, A., Oberhauser, V. & von Lintig, J. The role of 11-cis-retinyl esters in vertebrate cone vision. *FASEB J* **29**, 216-26 (2015).
2. Kane, M.A. & Napoli, J.L. Quantification of endogenous retinoids. *Methods Mol Biol* **652**, 1-54 (2010).
3. Landers, G.M. & Olson, J.A. Rapid, simultaneous determination of isomers of retinal, retinal oxime and retinol by high-performance liquid chromatography. *J Chromatogr* **438**, 383-92 (1988).
4. Zonta, F. & Stancher, B. High-performance liquid chromatography of retinals, retinols (vitamin A1) and their dehydro homologues (vitamin A2): improvements in resolution and spectroscopic characterization of the stereoisomers. *J Chromatogr* **301**, 65-75 (1984).

Table S2. Primer sequences

Primer	Sequence
**Degenerate PCR primers used to amplify *Petromyzon marinus CYP27C1***	
Cyp27c1_327_degen_F	AAGACCCACTTCGGCccncarttygt
Pm_3'_A_Cyp27c1	TGAGAGACGGTCCACAAAGTG
**Gene specific primers used to amplify 5’ and 3’ ends of *Petromyzon marinus CYP27C1***	
Pm_Cyp27c1_1143_F	ACTACAGCACCTCGCACGAC
Pm_Cyp27c1_413_F	CATGTTCAAGACCACCATGTA
Pm_Cyp27c1_1421R	GCGCCAGGTGAATCTCCAAC
Pm_ Cyp27c1_413_R	CACGTGAATCTCGCTGAAG
Pm_ Cyp27c1_119_R	CCATGGCGAACTTGAAGA
**qPCR primers used to quantify expression of *Petromyzon marinus CYP27C1***	
Pm_q_Cyp27c1_861_f	AGGTTGTACCCGGTGTT
Pm_q_Cyp27c1_861_r	GTGGCACATAGCCAGTTG
Pm_q_GAPDH_815_f	TGGGATACACCGAGGATTT
Pm_q_GAPDH_815_r	GACGAACTGCTTGTTGAGAG
**Primers used to generate in situ hybridization probes against *CYP27C1* and *RHO***	
Pm_Cyp27c1_5’_A	AACGGGCTCCTCTTCAAGTT
Pm_Cyp27c1_3'_A	TGAGAGACGGTCCACAAAGTG
Pm_Rho_249F	CCTCTTTATGGTCCTCTTCGGCTTC
Pm_Rho_839R	CCCTGGTGGGTGAAGATGTAGAAG
